# Outcomes of Heart Failure Related Hospitalizations During the COVID-19 Pandemic

**DOI:** 10.7759/cureus.36935

**Published:** 2023-03-30

**Authors:** Abdulmajeed Alharbi, Halah Alfatlawi, Abdelrhman Mohamed, Mohammed Mhanna, Mona Mahmoud, Rawnag Elsheik, George Moukarbel, Ragheb Assaly

**Affiliations:** 1 Internal Medicine, University of Toledo, Toledo, USA; 2 Internal Medicine, The University of Toledo Medical Center, Toledo, USA; 3 Cardiology, University of Iowa, Iowa, USA; 4 Cardiology, The University of Toledo Medical Center, Toledo, USA; 5 Pulmonary/Critical Care Medicine, The University of Toledo Medical Center, Toledo, USA

**Keywords:** covid-19, heart failure hospitalization, heart failure with preserved ejection fraction, heart failure with reduced ejection fraction, heart failure

## Abstract

Background: The incidence and prevalence of heart failure (HF) in the United States has steadily increased in the past few decades. Similarly, the United States has experienced an increase in HF-related hospitalizations which has added to the burden of a resource-stretched healthcare system. With the emergence of the coronavirus disease 2019 (COVID-19) pandemic in 2020, hospitalizations due to the COVID-19 infection sky-rocketed further exacerbating the burden on both patient health and the healthcare system. The focus of this study is to examine how a secondary COVID-19 diagnosis affects the outcome of HF patients, and how a pre-existing diagnosis of heart failure impacts the outcomes of patients hospitalized with COVID-19 infection.

Methods: This was a retrospective observational study of adult patients hospitalized with heart failure and COVID-19 infection in the United States in the years 2019 and 2020. Analysis was conducted using the National Inpatient Sample (NIS) database of the Healthcare Utilization Project (HCUP). The total number of patients included in this study from the NIS database 2020 was 94,745. Of those, 93,798 had heart failure without a secondary diagnosis of COVID-19; 947 had heart failure along with a secondary diagnosis of COVID-19. The primary outcome of our study was in-hospital mortality, length of stay, total hospital charges and time from admission to right heart catheterization, which were compared between the two cohorts.

Results: Our main study findings are that mortality in HF patients with secondary diagnosis of COVID-19 infection was not statistically different compared to those who were without a secondary diagnosis of COVID-19. Our study findings also showed that length of stay (LOS) and hospital costs in HF patients who had a secondary diagnosis of COVID-19 were not statistically different compared to those who did not have the secondary diagnosis. Time from admission to right heart catheterization (RHC) in HF patients who had a secondary diagnosis of COVID-19 was shorter in heart failure with reduced ejection fraction (HFrEF) but not in heart failure preserved ejection fraction (HFpEF) compared to those without secondary diagnoses of COVID-19. Finally, when evaluating hospital outcomes for patients admitted with COVID-19 infection, we found that inpatient mortality increased significantly when they had a pre-existing diagnosis of heart failure.

Conclusion: The COVID-19 pandemic significantly impacted hospitalization outcomes for patients admitted with heart failure. The time from admission to right heart catheterization was significantly shorter in patients admitted with heart failure reduced ejection fraction who also had a secondary diagnosis of COVID-19 infection. When evaluating hospital outcomes for patients admitted with COVID-19 infection, we found that inpatient mortality increased significantly when they had a pre-existing diagnosis of heart failure. Length of hospital stay and hospital charges also were higher for patients with COVID-19 infection who had pre-existing heart failure. Further studies should focus not just on how medical comorbidities like COVID-19 infection, affect outcomes of heart failure but also on how overall strains on the healthcare system, such as pandemics, may affect the management of conditions such as heart failure.

## Introduction

Heart failure (HF) was designated as an epidemic in 1997 due to an observed exponential increase in hospitalization rate [1]. In a 2021 statistical analysis, the American Heart Association (AHA) estimated the prevalence of HF in the US to be ~ 1.8% (6 million) of the population [2]. From 2014 to 2017, there was a 26% increase in observed HF-related hospitalizations. The rate of hospitalization is considered a strong predictor of readmission as well as mortality [3]. Since 2012, mortality caused by HF has increased from 275,000 in 2009 to 310,000 in 2014. This is particularly evident in the elderly population due to a higher prevalence of comorbidities [4]. The public health and economic burden add further highlights to the importance of careful investigation and management.

The coronavirus disease 2019 (COVID-19) caused by severe acute respiratory syndrome coronavirus 2 (SARS-CoV-2) spread rapidly by the end of 2019, creating a global health emergency. The spread of this virus further exacerbated the public health burden of HF [5]. Studies that compared HF admissions before and after the first wave of the COVID-19 pandemic have shown decreased HF-related admissions and possibly increased mortality. Resource allocation, the need to prioritize patients with higher life expectancy, lower availability of specialists, and reluctance to seek medical care, were some of the driving factors for the disparity seen during the pandemic [6,7]. 

Patients living with HF were particularly vulnerable to infection due to reduced immunity and hemodynamic ability to cope with the stress of severe infection [8]. Furthermore, SARS-CoV-2 enters cells through the angiotensin-converting enzyme 2 (ACE-2) receptor. The renin-angiotensin-aldosterone (RAAS) pathway plays a vital role in the pathogenesis of HF. This makes HF patients maintained on RAAS blockers possibly susceptible to severe COVID-19 complications [6]. Additionally, the management of HF patients with COVID-19 was challenging due to the similarities in clinical presentation, the ability of COVID-19 to interact with HF medications, and difficulty in performing interventional procedures [9]. 

Accordingly, since the onset of the pandemic, many studies have investigated the relationship between a COVID-19 diagnosis and HF. In a meta-analysis and systemic review that looked at a 21,640-patient sample from 18 studies, it was shown that patients with pre-existing HF that developed COVID-19 had a higher mortality rate than COVID-19 patients with no prior HF diagnosis [10]. Here we present a nationwide retrospective observational study of a total of 94,745 HF patients. The focus of this study is to examine how a secondary COVID-19 diagnosis affects the outcome of HF patients.

## Materials and methods

Study design

This was a retrospective observational study of adult patients hospitalized with a primary diagnosis of heart failure and a secondary diagnosis of COVID-19 infection in the United States in the year 2020. The study aims to estimate the impact of the COVID-19 pandemic on the outcomes of patients admitted with heart failure. This study also aimed to assess the outcomes of patients admitted with COVID-19 infection who had a pre-existing diagnosis of heart failure.

Data source and sample

For data analysis, we utilized one of the largest inpatient databases in the United States, the National Inpatient Sample (NIS) which contains discharge data from a 20% stratified sample of United States hospitalizations nationwide. The NIS uses the tenth revision of the International Classification of Diseases (ICD10-CM) for codes for diagnosis. We included codes for primary diagnosis of congestive heart failure (ICD10-CM I1504), Heart failure reduced ejection fraction (ICD10-CM I1502), heart failure preserved ejection fraction (ICD10-CM I1503) and a code for co-morbid COVID-19 infection (ICD10-CM U071). We excluded patients with diagnosis of shock (ICD10-CM R571, R578, R579, R6521) and patients requiring mechanical ventilation (ICD10-CM T884XXD, T88DXXS, T884XXA, T884, 5A1522G).

Variables of interest and study outcomes

The primary outcome of our study was in-hospital mortality, length of stay, total hospital charges and time from admission to right heart catheterization (RHC), which were compared between the two cohorts which is with or without secondary diagnosis of COVID-19. The secondary outcome of our study was to evaluate the impact of preexisting heart failure on COVID-19 patients in terms of inpatient mortality, length of stay and total hospital charges. The exposure of interest was COVID-19 infection. Potential confounders that were adjusted for included age in years, female sex, race, median income in patient’s zip code, patient comorbidities measured using Charlson Comorbidity Index (CCI) for administrative data, hospital region, hospital location and teaching status, hospital bed size and primary expected payer.

Statistical analysis

In accordance with the Healthcare Cost and Utilization Project data use agreement, we did not report variables containing a small number of observations (≤10) that could pose risk of patient identification or data privacy violation.

Statistical analysis was performed using STATA version 17.0 software (StataCorp., College Station, TX) [11]. Data was expressed as percentages for categorical variables and mean ± SD for continuous variables. Continuous variables were compared using Student's t-test and categorical variables were compared using the chi-square test. Univariate regression analysis was used to calculate unadjusted odds ratios for the primary and secondary outcomes. Multivariate regression analysis was used to adjust for the potential confounders and calculate adjusted odds ratios (aOR). A logistic regression model was used for binary outcomes and linear regression for continuous outcomes. The models were built by including the variables that were associated with the outcome of interest on univariable regression analysis, with a cut-off p = 0.02. All p values were 2-sided, with 0.05 as a threshold for statistical significance.

## Results

Population demographics

The total number of admissions included in this study from the NIS database 2020 was 94,745. Of those, 93,798 had heart failure without a secondary diagnosis of COVID-19; 947 had heart failure along with a secondary diagnosis of COVID-19. Table 1 shows patient demographics for the two groups, in addition to hospital characteristics. The mean age for patients with heart failure and with a secondary diagnosis of COVID-19 infection was 65.2 years old, with a mean age of 66.5 for those with heart failure without COVID-19. Males made up 55% of those with heart failure with no secondary diagnosis of COVID-19 and 69% of patients hospitalized with heart failure and a secondary diagnosis of COVID-19. Twenty-one percent of patients with heart failure without COVID-19 as a secondary diagnosis were obese, and 19% had diabetes mellitus with chronic complications. For patients hospitalized with heart failure along with COVID-19, 16% were obese, and 31% of patients with diabetes mellitus with chronic complications. Of patients hospitalized with heart failure and a secondary diagnosis of COVID-19, 15% had complicated hypertension, compared to 23% of those with heart failure without COVID-19 infection. Other comorbidities such as peripheral vascular disease, chronic pulmonary disease, drug abuse, and alcohol abuse are accounted for in Table 1 with respected p values. Hospital characteristics and socio-economic information are also included in Table 1.

**Table 1:** Baseline demographic characteristics of patients Patients included are with a primary diagnosis of heart failure, with and without a history of COVID-19 infection. P-value ≤ 0.05 indicates significance. HF: Heart Failure

Variable	HF with COVID-19	HF without COVID-19	p-value
Number of Patients	947 (1.0%)	93798 (99.0%)	
Age mean (years)	65.2	66.5	<0.001
Female Gender (%)	31%	45%	<0.001
Race (%)			0.26
Caucasian	57%	73%	
African American	16.7%	14%	
Hispanic	18.7%	7.1%	
Asian or Pacific Islander	2.0%	1.9%	
Native American	1.5%	0.09%	
Others	3.4%	2%	
Median Income in patient zipcode (%)			0.50
$1-$47,999	34%	31%	
$48,000-$60,999	24%	30%	
$61,000-81,999	22%	22%	
≥$82,000	19%	17%	
Charlson comorbidity index (%)			
0	0	0	
1	16%	19%	
2	22%	25%	
3 or more	62%	56%	
Hospital Region			0.03
Northeast	25%	17%	
Midwest	23%	24%	
South	26%	35%	
West	25%	24%	
Hospital Bed size (%)			0.23
Small	22%	27%	
Medium	23%	24%	
Large	55%	48%	
Hospital Urban Location (%)			.02
Rural	13%	17%	
Urban nonteaching	11%	18%	
Urban teaching	77%	67%	
Primary expected payer (Insurance )			0.83
Medicare	61%	61%	
Medicaid	19%	18%	
Private Insurance	15%	17%	
Self-Pay	5%	5%	
Comorbidities			
Drug Abuse (%)	8.6%	17%	0.001
Hypertension, complicated (%)	15%	23%	0.01
Hypertension, uncomplicated (%)	7.0%	6.5%	0.79
Diabetes with chronic complications (%)	31%	19%	<0.01
Diabetes without chronic complications (%)	11%	10%	0.67
Alcohol Abuse (%)	8.5%	6.4%	0.33
Obesity (%)	16%	21%	0.13
Peripheral Vascular Disease (%)	4.9%	7.7%	0.20
Chronic Pulmonary Disease (%)	30%	36%	0.22

Inpatient mortality outcome

Patients who had heart failure with reduced ejection fraction (HFrEF) with a secondary diagnosis of COVID-19 had a higher inpatient mortality than patients without a secondary diagnosis of COVID-19 (aOR 1.13 CI: 0.51-2.49, p 0.76). Patients who had heart failure preserved ejection fraction (HFpEF) with a secondary diagnosis of COVID-19 had lower inpatient mortality than patients without a secondary diagnosis of COVID-19 infection (aOR 0.41 CI: 0.05-3.43, p 0.41). Mortality outcomes for both HFrEF and HFpEF and COVID-19 were not statistically significant with p-values greater than (0.05). Age, gender, race, Charlson comorbidity Index (CCI) were kept constant. These results are shown in Table 2.

**Table 2:** Impact of COVID-19 infection on patient outcomes Impact of COVID-19 infection on inpatient mortality, length of stay, hospital charges, and time from admission to PCI in adults hospitalized with heart failure. A p-value ≤ 0.05 indicates significance. HFrEF- Heart failure reduced ejection fraction; HFpEF- Heart failure preserved ejection fraction; RHC- Right heart catheterization; PCI-Primary Coronary Intervention

	Adjusted Odds Ratio and Mean	95% Confidence Interval	p-value
HFrEF (63460)			
Mortality	1.13	0.51- 2.49	0.76
Length of stay	-0.04	-1.68- 1.58	.95
Hospital charges	- $19413.60	- $48954.9 - $10127.86	0.19
Time from admission to RHC	-2.39	-4.34- -0.44	0.02
HFpEF (27315 )			
Mortality	0.41	0.05- 3.43	0.41
length of stay	-0.24	-1.60- 1.10	0.71
Hospital charges	-$8035.40	- $27664.3- $11593.4	0.42
Time from admission to RHC	-1.07	-4.32- -2.16	0.05

Length of stay (LOS)

We compared length of stay for patients hospitalized with HF and with or without a secondary diagnosis of COVID-19. Length of stay comparisons were not statistically significant with p-values greater than 0.05. For patients with HFrEF and HFpEF, the mean length of stay was lower for patients with a secondary diagnosis of COVID-19, versus those without (HFrEF LOS: -0.04 Days CI: -1.68-1.58, p 0.95; HFpEF LOS: -0.24 Days, CI: -1.60-1.10, p 0.71). These results are shown in Table 2.

Hospital cost and resource utilization

When assessing costs, there were lower costs associated with hospital stays for patients with heart failure and a secondary diagnosis of COVID-19 infection when compared to those without a secondary diagnosis of COVID-19 infection, however, hospital charge comparisons were not statistically significant with p-values greater than 0.05 (HFrEF $19413.6 CI $48954.9 - 10127.86, p 0.19; HFpEF $8035.4 CI $27664.3 - 11593.4, p 0.42). These results are shown in Table 2.

Time from admission to right heart catheterization

We compared the time from admission to right heart catheterization (RHC) for our patient populations with heart failure with and without a secondary diagnosis of COVID-19 infection. Patients with heart failure (either HFrEF or HFpEF) and with a secondary diagnosis of COVID-19 had a lower mean time from admission to RHC (HFrEF -2.39 days CI: 4.34- -0.44, p 0.016; HFpEF -1.07 CI: -4.32- -2.16, p 0.51). When we compare the data for the year 2019, the pre-pandemic period, to the year 2020, the time from admission to RHC was higher pre-pandemic, with an average time of 3.66 days in 2019 for HFrEF compared to 3.5 days in 2020, and 4.04 days in 2019 for HFpEF compared to 3.9 in 2020. These results are shown in Table 3.

**Table 3:** Primary outcomes for patients admitted with heart failure in the year 2020 versus 2019. HFrEF- Heart failure reduced ejection fraction; HFpEF- Heart failure preserved ejection fraction; RHC- Right heart catheterization

	2020	2019	Adjusted Odds ratio/ Mean	95% Confidence Interval	p-value
HFrEF	63460	112864			
Inpatient Mortality	2450 (3.8%)	2699 (3.5%)	1.12	0.96- 1.26	0.10
Length of Stay (in days)	5.6	5.5	0.06	-0.30- 0.43	0.74
Time from Admission to RHC	3.5	3.66	-0.17	-0.52- 0.18	0.35
HFpEF	27315	33239			
Inpatient Mortality	874 (3.2%)	1004 (3.0%)	1.08	0.87- 1.34	0.45
Length of Stay	5.2 days	5.2 days	-0.006	-0.23- 0.21	0.95
Time from Admission to RHC	3.9	4.04	-0.15	-0.88- 0.56	0.67

Impact of the COVID-19 pandemic on HFrEF and HFpEF compared to pre-pandemic

Table 3 compares the pre-pandemic years 2019-2020, the COVID-19 pandemic 2020, and compares outcomes such as inpatient mortality, length of stay and hospital charges for heart failure hospitalizations. Among patients with HFrEF, inpatient mortality, length of stay, and hospital charges were higher during the pandemic. For patients with HFpEF, inpatient mortality was also higher during the pandemic, length of stay was the same with a mean of 5.2 days and hospital charges were higher during the pandemic compared to data from the year 2019, before the pandemic. These results are shown in Table 3.

Impact of pre-existing HF on a patient admitted with a primary diagnosis of COVID-19 infection

For patients who were admitted with a primary diagnosis of COVID-19 infection in the year 2020, we assessed the effect of having a pre-existing heart failure diagnosis. We compared inpatient mortality; length of hospital stays and hospital charges between those who were admitted with COVID-19 infection who also had heart failure and those without a history of heart failure. Results are shown in Table 4. Inpatient mortality was significantly higher for patients admitted with COVID-19 infection with a secondary diagnosis of heart failure (aOR 1.13, CI 1.08-1.19 p <0.01). Length of stay was longer and hospital charges were higher when patients who were admitted with a primary diagnosis of COVID-19 infection had a secondary diagnosis of heart failure, although both were not statistically significant (mean length of stay 0.04 days CI -0.09 - 0.18, p 0.59; mean hospital charges $948.19 CI -1699.45 - 3595.84, p 0.43). These results are shown in Table 4.

**Table 4:** Impact of pre-existing heart failure on patients admitted with a primary diagnosis of COVID-19 infection.

	Adjusted Odds Ratio/Mean	95% Confidence Interval	p-value
Inpatient Mortality	1.13	1.08- 1.19	≤ 0.01
Length of Stay (in days)	0.04	-0.09- 0.18	0.59
Hospital Charges	$ 948.19	- $1699.45- $3595.84	0.43

## Discussion

In this large nationwide observational study, and to the best of our knowledge this is the largest analysis to date, we explored the outcomes of HF patients in this patient population during the first year of the COVID-19 pandemic. Our main study findings are that mortality in HF patients with secondary diagnosis of COVID-19 was not statistically different compared to those who were without a secondary diagnosis of COVID-19. Our study findings also showed that length of stay (LOS) and hospital costs in HF patients who had a secondary diagnosis of COVID-19 were not statistically different compared to those who did not have the secondary diagnosis. Time from admission to right heart catheterization (RHC) in HF patients who had a secondary diagnosis of COVID-19 was shorter in HFrEF but not in HFpEF compared to those without secondary diagnoses of COVID-19. Lastly, when evaluating hospital outcomes for patients admitted with COVID-19 infection, patients that had a pre-existing diagnosis of heart failure had higher inpatient mortality rates and longer lengths of stay than those without pre-existing heart failure.

The relation between heart failure and COVID-19 is bidirectionally interrelated in both direct and indirect ways. Bhatt et al. observed that in hospitalized patients with HF, having COVID-19 was associated with greater odds of in-hospital mortality (24.2%) as compared to those hospitalized only for acute HF exacerbation (2.6%) [12]. Alvarez-Garcia et al. observed that in patients hospitalized for COVID-19, HF patients had an increased risk of in-hospital mortality (40.0% vs. 24.9%) as well as an average longer length of stay, eight days vs six days (p<0.001) [13]. Tomasoni et al. observed that in 692 consecutive patients hospitalized for COVID-19, there was a greater percentage of in-hospital mortality in patients with HF vs no history of HF [10]. Of all in-hospital mortality, acute heart failure was the cause of death in 9.1% of the patients, a greater portion being from the patients with pre-existing HF (33.3%); however, overall, 24 patients hospitalized with COVID-19 without pre-existing HF died from acute HF (p<0.001) [10], similar findings were reported by two other studies [14,15].

A large observational study conducted by Fox et al., analyzed 27,427 emergency department visits or hospitalizations from a 12-hospital health system to examine changes in treatment (right and left-heart catheterization), and outcomes for patients with heart failure (HF) during the COVID-19 pandemic compared with pre-COVID-19. They reported that there was no difference in the rate of RHC, length of stay, or mortality [16].

On the other hand, a meta-analysis by Yonas et al. observed that seven studies reported a statistically significant difference in mortality between patients with COVID-19 with and without pre-existing HF [17]. This is similar to our study findings of increased inpatient mortality for patients admitted with COVID-19 with preexisting heart failure. 

COVID-19 infection may alter the course of heart failure and other cardiac conditions through activation of the inflammatory cascade, among other numerous potential pathways involved in the pathogenesis of the infection and the complications resulting from it. The stress of the infection may result in new-onset heart failure, or an acute exacerbation of pre-existing heart failure and a number of studies have observed the effects of COVID-19 on outcomes of either pre-existing or new-onset heart failure [10,17].

Limitations

There are some limitations in this study. First, we used a database to select patients who had specific diagnoses. Coding errors and documentation errors may be present. We anticipate this to be small in number and insignificant. Second, patient-specific data, such as individual lab results, oncologic status, medications, imaging results, that would be expected to influence outcomes were not accounted for. Third, while inpatient mortality was accounted for in this study, mortality after discharge from the hospital and mortality without hospitalization were not accounted for and were not available in the database. Finally, there may be selection bias or unmeasured bias in any observational study including this meta-analysis. We completed a multivariable analysis to reduce allocation bias.

## Conclusions

The COVID-19 pandemic significantly impacted hospitalization outcomes for patients admitted with heart failure. This study provides a nationwide retrospective study of a large number of patients with heart failure during the COVID-19 pandemic to evaluate the effect of the pandemic on hospital outcomes. The time from admission to right heart catheterization was significantly shorter in patients admitted with heart failure reduced ejection fraction who also had a secondary diagnosis of COVID-19 infection. When evaluating hospital outcomes for patients admitted with COVID-19 infection, we found that inpatient mortality increased significantly when they had a pre-existing diagnosis of heart failure. Length of hospital stay and hospital charges also were higher for patients with COVID-19 infection who had pre-existing heart failure than for those without pre-existing heart failure. Further studies should focus not just on how medical conditions, such as COVID-19 infection, affect outcomes of heart failure but also on how overall strains on the healthcare system, such as pandemics, may affect the management of conditions such as heart failure.
